# Supplementary material for: The efficacy and safety of roxadustat for the treatment of anemia in non-dialysis dependent chronic kidney disease patients: An updated systematic review and meta-analysis of randomized clinical trials
Source: PLoS One. 2022 Apr 1;17(4):e0266243. doi: 10.1371/journal.pone.0266243 (PMC8974992; doi:10.1371/journal.pone.0266243)
Supplement: S1 Fig — (DOCX) [file pone.0266243.s002.docx]

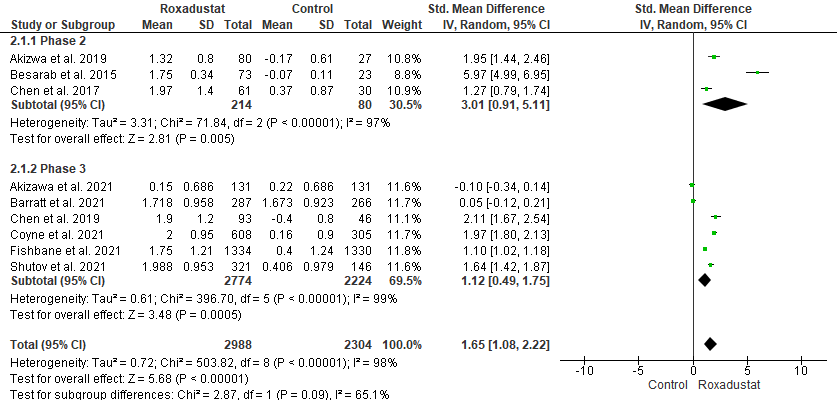


Figure S1 Forest plot of the effect of trial phase on hemoglobin level

CI: confidence interval; M-H: Mantel-Haenszel; df: degrees of freedom; I^2^, I-squared.
