# Supplementary figures and images for: The efficacy and safety of roxadustat for the treatment of anemia in non-dialysis dependent chronic kidney disease patients: An updated systematic review and meta-analysis of randomized clinical trials
Source: PLoS One. 2022 Apr 1;17(4):e0266243. doi: 10.1371/journal.pone.0266243 (PMC8974992; doi:10.1371/journal.pone.0266243)

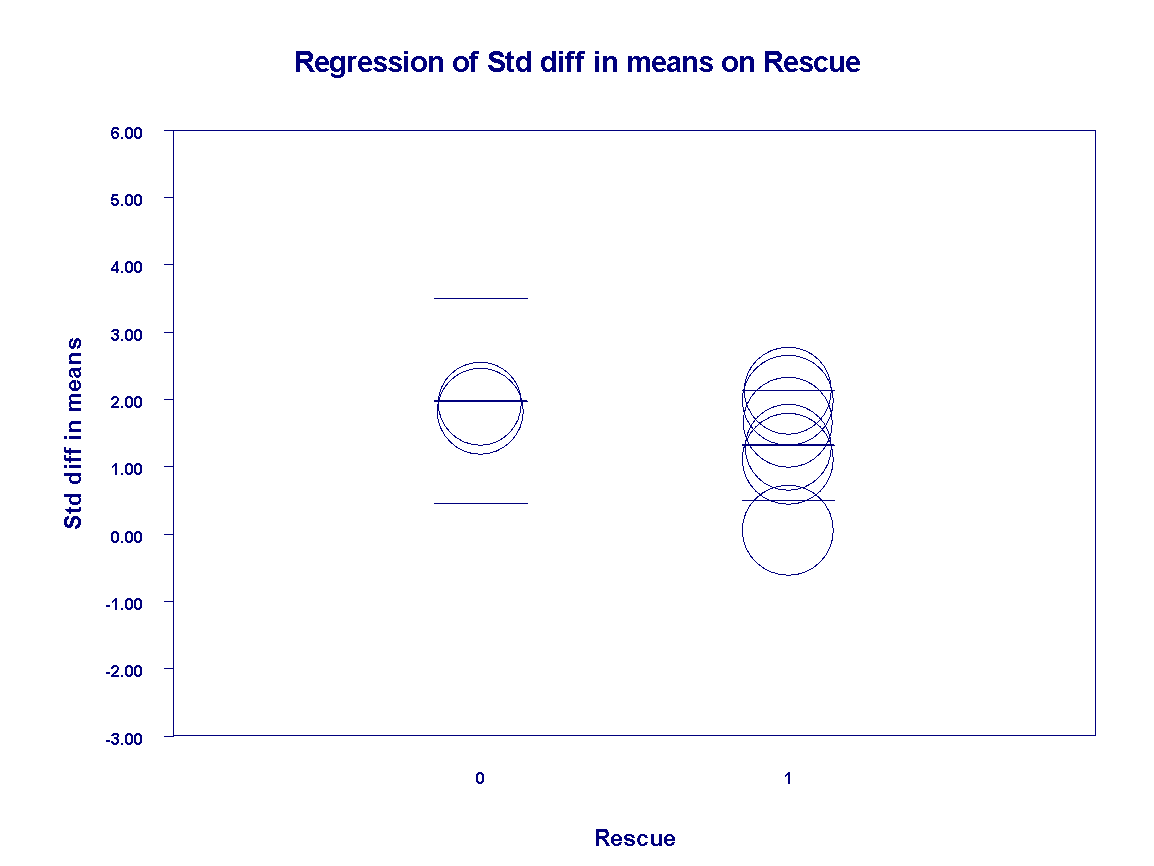


Figure S9 Regression of standardized difference in means on Rescue

Supplement: S9 Fig — (DOCX) [file pone.0266243.s010.docx]

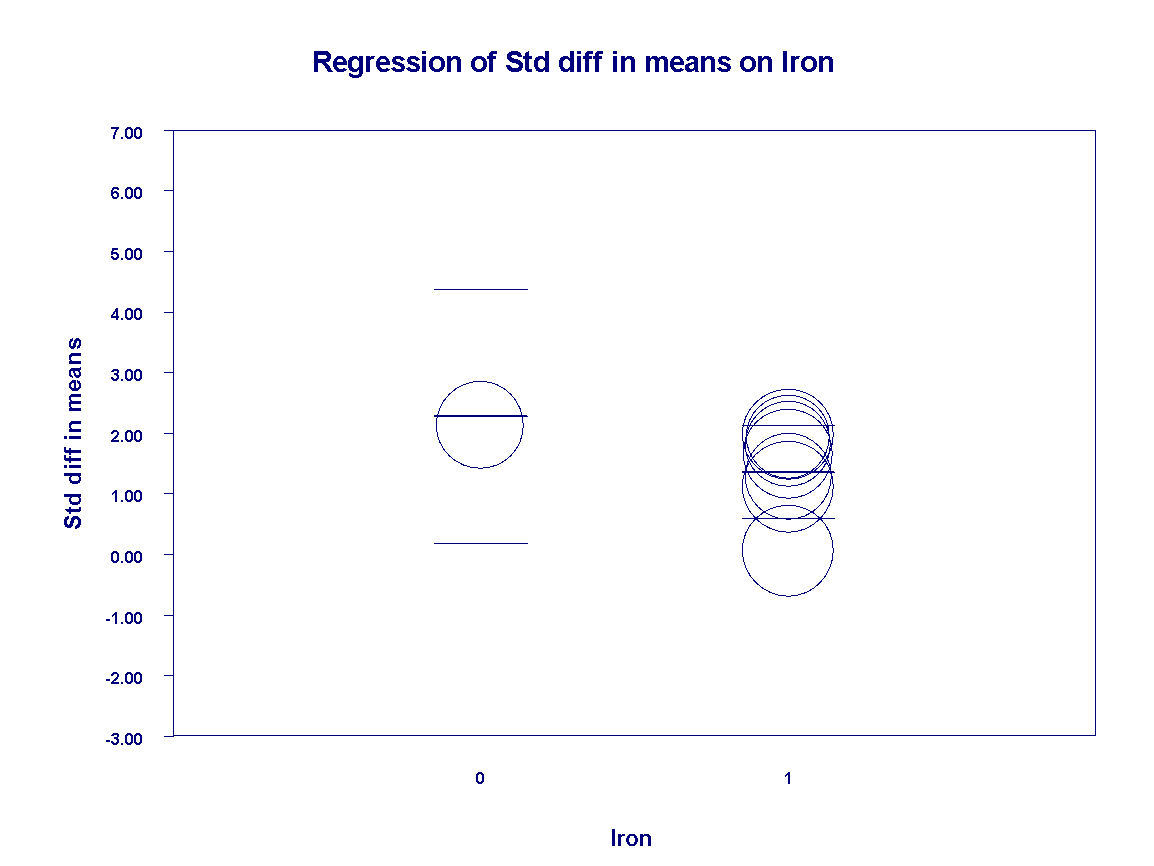


Figure S10 Regression of standardized difference in means on iron

Supplement: S10 Fig — (DOCX) [file pone.0266243.s011.docx]
