# Supplementary material for: The efficacy and safety of roxadustat for the treatment of anemia in non-dialysis dependent chronic kidney disease patients: An updated systematic review and meta-analysis of randomized clinical trials
Source: PLoS One. 2022 Apr 1;17(4):e0266243. doi: 10.1371/journal.pone.0266243 (PMC8974992; doi:10.1371/journal.pone.0266243)
Supplement: S1 Table — (DOCX) [file pone.0266243.s012.docx]

| PubMed | (Roxadustat OR ASP1517 OR FG4592 OR "FG-4592") AND (kidney OR renal) AND (Anemia) | All Field | 104 |
| --- | --- | --- | --- |
| Web Of Science | (Roxadustat OR ASP1517 OR FG4592 OR "FG-4592") AND (kidney OR renal) AND (Anemia) | Topic | 150 |
| Scopus | (Roxadustat OR ASP1517 OR FG4592 OR "FG-4592") AND (kidney OR renal) AND (Anemia) | Title, Abstract, Keywords | 159 |
| Cochrane | ((Roxadustat) OR (ASP1517) OR (FG4592) OR (FG-4592)) AND ((kidney) OR (renal)) AND ((Anemia)) | Title, Abstract Keyword | 113 |
| Embase | (roxadustat OR asp1517 OR fg4592 OR 'fg 4592') AND (kidney OR renal) AND anemia | All Field | 256 |
| Google Scholar | with all of the words: Roxadustat OR FG4592 OR "FG-4592"  with at least one of the words: kidney anemia  In the title of the article |  | 126 |

Table S1 Search terms and results in different databases
