# Supplementary material for: The efficacy and safety of roxadustat for the treatment of anemia in non-dialysis dependent chronic kidney disease patients: An updated systematic review and meta-analysis of randomized clinical trials
Source: PLoS One. 2022 Apr 1;17(4):e0266243. doi: 10.1371/journal.pone.0266243 (PMC8974992; doi:10.1371/journal.pone.0266243)
Supplement: S2 Table — (DOCX) [file pone.0266243.s013.docx]

| Outcome | No. of  participants (Roxadustat/Control) | No. of  trials | Quantitative data synthesis | | | | Heterogeneity analysis | | |
| --- | --- | --- | --- | --- | --- | --- | --- | --- | --- |
|  |  |  | SMD | 95% CI | Z value | p-value | df | p-value | I2 (%) |
| Hemoglobin | | | | | | | | | |
| All studies | 2988/2304 | 9 | 1.65 | [1.08, 2.22] | 5.68 | 0.00001 | 8 | 0.00001 | 98 |
| Omitting Akizawa et al. 2021 | 2857/2173 | 8 | 1.87 | [1.29, 2.45] | 6.32 | 0.00001 | 7 | 0.00001 | 98 |
| Omitting Akizwa et al. 2019 | 2908/2277 | 8 | 1.62 | [1.01, 2.22] | 5.22 | 0.00001 | 7 | 0.00001 | 99 |
| Omitting Barratt et al. 2021 | 2701/2038 | 8 | 1.85 | [1.28, 2.42] | 6.40 | 0.00001 | 7 | 0.00001 | 98 |
| Omitting Besarab et al. 2015 | 2915/2281 | 8 | 1.23 | [0.69, 1.78] | 4.47 | 0.00001 | 7 | 0.00001 | 98 |
| Omitting Chen et al. 2017 | 2927/2274 | 8 | 1.70 | [1.09, 2.32] | 5.43 | 0.00001 | 7 | 0.00001 | 99 |
| Omitting Chen et al. 2019 | 2895/2258 | 8 | 1.59 | [0.99, 2.20] | 5.17 | 0.00001 | 7 | 0.00001 | 99 |
| Omitting Coyne et al. 2021 | 2380/1999 | 8 | 1.61 | [1.00, 2.22] | 5.18 | 0.00001 | 7 | 0.00001 | 98 |
| Omitting Fishbane et al. 2021 | 1654/974 | 8 | 1.78 | [0.96, 2.59] | 4.27 | 0.0001 | 7 | 0.00001 | 99 |
| Omitting Shutov et al. 2021 | 2667/2158 | 8 | 1.66 | [1.03, 2.30] | 5.11 | 0.00001 | 7 | 0.00001 | 99 |

Table S2 Meta-analysis of the primary outcomes and sensitivity analysis

CI: confidence interval; df: degrees of freedom; Std: standardized mean difference; I^2^, I-squared
